# Association of* NLRP1* and* NLRP3* Polymorphisms with Psoriasis Vulgaris Risk in the Chinese Han Population

**DOI:** 10.1155/2018/4714836

**Published:** 2018-04-03

**Authors:** Pei Yu, Siyu Hao, Hewei Zheng, Xueying Zhao, Yuzhen Li

**Affiliations:** ^1^Department of Dermatology, The Second Affiliated Hospital of Harbin Medical University, Harbin 150081, China; ^2^Department of Bioinformatics, Harbin Medical University, Harbin 150001, China

## Abstract

**Aim:**

To clarify the association between the single nucleotide polymorphisms (SNPs) in the* NLRP1* and* NLRP3* and Psoriasis Vulgaris (PsV) in the Chinese Han population.

**Methods:**

We genotyped eight SNPs, four from* NLRP1* (rs8079034, rs11651270, rs11657747, and rs878329) and* NLRP3* (rs7512998, rs3806265, rs10754557, and rs10733113) each in 540 patients with PsV and 612 healthy controls in the Chinese Han population using an improved multiplexed ligation detection reaction (iMLDR) method. The genotype and haplotype frequencies were analyzed using a case-control study design.

**Results:**

We identified two SNPs, rs3806265 and rs10754557, in* NLRP3* that were significantly associated with PsV. The genotype distribution of the rs3806265 SNP was significantly different between cases and controls (*p* = 0.0451; OR = 0.791; 95% CI = 0.627–0.998). In the recessive model, the genotype distribution of the rs10754557 SNP was significantly different between cases and controls (*p* = 0.0344; OR = 1.277; 95% CI = 0.987–1.652). The haplotype analysis of rs3806265 and rs10754557 also presented a significant association of TA haplotype with PsV (*χ*^2^ = 4.529; *p* = 0.033).

**Conclusion:**

NLRP3 may play a role in PsV susceptibility in the Chinese Han population.

## 1. Introduction

Psoriasis is a common, immune-mediated, chronic inflammatory skin disease that causes red, flaky patches of skin [1], which affects approximately 2% of the general population [2]. Although its etiology is not fully understood, psoriasis has been established as a complex and multifactorial disease related to environmental and genetic factors [3]. The number of identified loci for European populations has increased to 63 and many functional networks and gene regulatory signals associated with psoriasis have been revealed by the largest GWAS meta-analysis for psoriasis patients to date in European populations [4]. The most accurate genetic prediction model to date involving 14 psoriasis susceptibility loci shows that these loci have the discriminating potential, being also associated with family history and age of onset [3]. These findings provide new insights into the pathogenesis of psoriasis and have greatly enhanced our understanding of the disease mechanisms. However, a substantial portion of information regarding the heritability of the disease remains unknown and further studies are required [3].

Members of the nucleotide-binding oligomerization domain-like receptor (NLR) family are the major components of inflammasomes [5], which are intracellular complexes assembled by proinflammatory caspase, NLR family members containing pyrin domain (NLRP), and the adaptor molecule apoptosis-associated speck-like protein [6]. Inflammasomes play a vital role in the maturation and secretion of proinflammatory cytokines [7], including interleukin-1*β* [6, 8, 9], which cause a wide variety of biological effects associated with infection, inflammation, and autoimmune processes by triggering the adaptive immune response [10]. NLRP1 and NLRP3 are the two most well-studied inflammasome-forming NLR family members [9]. These proteins might represent important links between the innate and the adaptive immune system in chronic inflammatory diseases [11] and have been strongly implicated in the pathogenesis of psoriasis [8, 9].

NLRP1 and NLRP3 expression has been observed in the lesional as well as nonlesional psoriatic epidermis [11]. The* NLRP1 *mRNA expression level was reported to be increased in the peripheral blood of patients with psoriasis [9], and the NLRP3 expression level was reported to be increased in the leucocytes and keratinocytes of psoriasis lesions, but not in the parakeratotic cells on the surface of psoriasis lesions [12]. The NLRP3 inflammasome complex and protein components were detected in both cultured and primary human keratinocytes [12, 13]. Therefore, NLRP1 and NLRP3 may play roles in the dysregulated innate immune response that is characteristic of psoriasis.

Different autoimmune diseases share susceptibility loci [14]. For example, the association analysis by Li et al. identified two common susceptibility loci shared by psoriasis and systemic lupus erythematosus (SLE) in the Chinese Han population [14]. Single nucleotide polymorphisms (SNPs) in the* NLRP1 *have been implicated in several complex autoimmune conditions such as juvenile idiopathic arthritis [15], rheumatoid arthritis (RA), vitiligo, autoimmune Addison's disease, and Type I Diabetes [16]. Moreover,* NLRP3* gene polymorphisms have been explored as possible predisposing factors for development of a variety of diseases associated with autoimmune and/or inflammatory responses, such as RA, Crohn's disease, SLE, celiac disease, AIDS, Alzheimer's disease, abdominal aortic aneurysms [17], atopic dermatitis [17, 18], and malignant melanoma [19].* NLRP1* and* NLRP3* polymorphisms have been associated with psoriasis in the European population [9, 12]; however, their association with psoriasis in other populations remains unexplored. Psoriasis has a considerable ethnic variation. Allelic and/or locus heterogeneity exists between different populations [5, 20]. Sun et al. identified six susceptibility loci in the Chinese population by adopting a multistage analysis strategy for several large samples from a Chinese and European population [21], which fully illustrated this point of view. Therefore, it is important to determine the genetic association between SNPs in NLRP1 and NLRP3 and susceptibility of PsV in the Chinese population. Accordingly, the aim of the current study was to investigate the association between SNPs in* NLRP1 *and* NLRP3 *and susceptibility of PsV in the Chinese Han population.

## 2. Materials and Methods

### 2.1. Study Subjects

In total, 540 patients diagnosed with PsV and 612 healthy individuals were recruited from outpatients of the 2nd Affiliated Hospital of Harbin Medical University (January 2013 to April 2015) in this study. All patients and healthy controls in this study were of Han Chinese descent (including first-, second-, and third-degree relatives). All the patients with PsV had at least two skin lesions. Diagnosis was based on evaluation of clinical features by at least two trained dermatologists at the 2nd Affiliated Hospital of Harbin Medical University and confirmed by skin biopsy at the same institution. The healthy controls were randomly recruited from patients visiting the same hospital for routine health examinations, who were confirmed to not be relatives of the patient group. Self-reported information from a standard questionnaire was used to collect demographic and other characteristics (severity, age of onset, and family history, including three levels of relatives) from the patients and to exclude controls with psoriasis or a family history of psoriasis, including three levels of relatives, or any other systemic, infectious, autoimmune, atopic, or malignant disease. None of the patients had arthritis.

All participants provided written informed consent. The study protocol was approved by the ethics committee of the 2nd Affiliated Hospital of Harbin Medical University and was conducted in full accordance with the Ethics Guidelines of the 1975 Declaration of Helsinki.

### 2.2. DNA Extraction

Peripheral venous blood samples (2 mL) were collected from each participant in tubes containing ethylenediaminetetraacetic acid. Genomic DNA for genotyping was prepared using standard extraction procedures (QIAamp DNA Blood Mini Kit, Qiagen, Germany) according to the manufacturer's recommendations and stored at −20°C.

### 2.3. SNPs Selection

**Figure 1:**
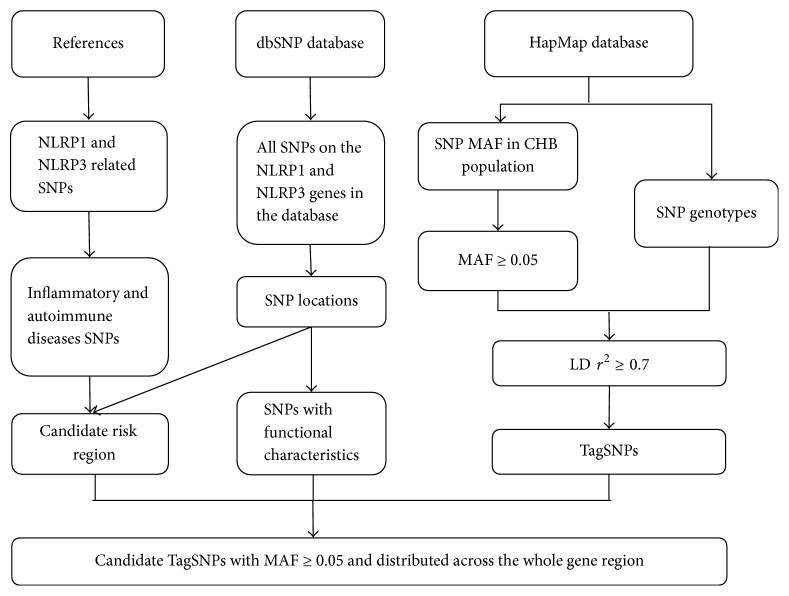
Flow chart depicting the process of SNP selection.

We refer to the method described by Zhao et al. to find reasonable SNPs [22]. Data on SNP loci for complete* NLRP1* and* NLRP3* sequences were obtained from the dbSNP database (https://www.ncbi.nlm.nih.gov/projects/SNP/), and SNP reference genotype data were retrieved from the NCBI HapMap database. First, we filtered all SNPs according to a HapMap SNP minor allele frequency ≥ 0.05 for the Han Chinese in Beijing (HCB) population. We then preferentially selected the SNPs, namely, rs8079034, rs11651270, rs11657747, rs878329, rs7512998, and rs10733113, according to their linkage relationship, frequencies, and positions, and their reported associations with immune and inflammatory disease. Following comprehensive analysis of the experimental conditions for genotyping, we ultimately selected four SNPs in* NLRP1* (rs8079034, rs11651270, rs11657747, and rs878329) and four SNPs in NLRP3 (rs7512998, rs3806265, rs10754557, and rs10733113) for our analysis. The specific process for selection is schematically represented by the SNP-screening flow chart (Figure 1).

### 2.4. Genotyping

Genotyping was performed using the iMLDR technique developed by Genesky Biotechnologies Inc. (Shanghai, China) as described previously [23].

Multiplex polymerase chain reaction (PCR) was used to amplify fragments including these eight SNP sites (see Tables S1 and S2 for the primer information). The 20 *μ*L PCR system consisted of 1 U HotStart Taq DNA polymerase (Qiagen, Germany), 1x GCI buffer, 3.0 mM Mg^2+^, 1 *μ*L primer mixture, 20 ng genomic DNA, and 0.3 mM dNTP (Generay Biotech). The PCR cycling program was as follows: 95°C for 2* * min; 11 cycles of 94°C for 20 s, decreasing to 65°C at 0.5°C increments per cycle for 40 s, and 72°C for 1.5 min; 24 cycles of 94°C for 20 s, 59°C for 30 s, and 72°C for 1.5 min; and a final extension at 72°C for 2 min, followed by holding at 4°C. The products were purified via digestion of 5 U shrimp alkaline phosphatase (Promega) and 2 U exonuclease I at 37°C for 1 h, followed by 75°C for 15 min. The ligation reaction was carried out in 10 *μ*L of a mixture comprising 1 *μ*L 10x ligation buffer, 0.25 *μ*L 80 U/*μ*L Taq DNA ligase (New England Biolabs), 0.4 *μ*L 5′ ligation primer mixture (1 *μ*M), 2 *μ*L purified PCR products, and 6 *μ*L ddH2O. The thermal conditions were as follows: 38 cycles of 94°C for 1 * *min, 56°C for 4 min, and holding at 4°C. The diluted ligation product (0.5 *μ*L) was mixed with 0.5 *μ*L Liz500 Size Standard and 9 *μ*L Hi-Di, denatured at 95°C for 5 min, and then loaded in the ABI3730XL sequencer. The raw data were analyzed using GeneMapper 4.1 software (Applied Biosystems, Foster City, CA, USA). The SNPs were identified according to different extension lengths of the 3′ terminal. We used different fluorescent labels of the allele-specific oligonucleotide probe to distinguish the specific alleles of each SNP.

Finally, to ensure the quality of the data, genotyping was performed by independent staff blinded to the case/control status of the samples, using ddH2O as the negative control for each reaction. A random DNA sample accounting for 5% of the total was genotyped again to determine the reproducibility.

### 2.5. Statistical Analysis

Demographic characteristics between the case and control groups (e.g., age, gender, stage, age of onset, severity, and family history) were evaluated by SPSS version 19.0. The distribution of detected genotypes, alleles, and conformation with Hardy-Weinberg equilibrium (HWE, evaluated at <0.001) were performed using Haploview 4.0. Four genetic models were adopted based on the allele frequencies of each locus in the cases to further evaluate differences in genotype distributions: additive, dominant, recessive, and heterozygous models. The allele and genotype frequencies were statistically compared between the cases and controls for each genetic model. The chi-squared test was used to establish the associations of* NLRP1 *and* NLRP3 *polymorphisms between cases and controls. Single SNP association analyses were conducted using SNPtest (wtccc) (http://mathgen.stats.ox.ac.uk/genetics_software/snptest/snptest.html). The association model (additive, dominant, recessive, and heterozygous) of each allele was tested using logistic regression.

The statistical significance was defined as *p* < 0.05 for a single test. Subsequently, we used the permutation method to perform the multiple test correction. For each SNP, we kept the population number of case and control groups unchanged, but disturbed the genotype 10,000 times to obtain 10,000 chi-square *p* values for the disturbed samples. Then, we defined the *p*′ value equivalent to the distribution of the original *p* calculated from the true data in the simulated *p* values. The linkage disequilibrium (LD), haplotype inference, and haplotype-based association analyses were carried out using Haploview tools. The LD (*r*^2^) threshold was 0.8 and *p* < 0.05 was identified as the statistically significant LD. Finally, we estimated the sample size to confirm the reliability of the data results.

## 3. Results

### 3.1. Samples and Description of the Genotyping Data

In total, 1152 subjects that participated in the study were successfully genotyped. The case and control groups were effectively age- and gender-matched (*p* = 0.20 and *p* = 0.24, resp.), and there was no significant difference (*p* > 0.05) between NLRP1 and NLRP3 genotypes according to age at disease onset (≤40 versus ≥40 years) or severity or family history. The blinded genotyping concordance rate was 100%, and no discrepancies were identified in the randomly selected (5%, *n* = 58) samples. The characteristics of the participants are provided in Table 1. In the case group, most PsV patients were in relatively mild condition (82.6% with PASI (psoriasis area and severity index) ≤ 10 and 17.4% with PASI ≥ 10).

In the 1152 quality control samples, the genotype reproducibility was 100%. No SNP revealed evidence of Mendelian genetic error, and none of the polymorphisms deviated from the HWE expectation in either cases or controls.

### 3.2. Single SNP Association Analysis

Initially, we selected 32 SNPs in NLRP1 with MAF ≥ 0.05 in CHB population, which contains 5 linkage disequilibrium modules. Combining with the SNPs mentioned in the literature and function characteristics of theses SNPs, we chose a representative SNP from each module, and finally 4 SNPs were identified. Similarly, we selected 116 SNPs in NLRP3 with a MAF ≥ 0.05 in CHB population, which contains 12 linkage disequilibrium modules. Combining with the SNPs mentioned in the literature and SNPs function characteristics, we chose a representative SNP from each module, and finally 4 SNPs were chosen.

The human NLRP1 gene is 83.1 kb long, located on chromosome 17p13.2. The human NLRP3 gene is 33.0 kb long, located on chromosome 1q44. The allele and genotype frequencies of the eight SNPs in patients and controls are summarized in Tables 2–4. For the rs3806265 SNP, the risk allele T was present in 56.5% of patients and 50.7% of controls. For the rs10754557 SNP, the risk allele A was present in 74.1% of patients and 69.1% of controls. The genotype distribution of the rs3806265 SNP was significantly different between the cases and controls (*p* = 0.0451; *p*′ = 0.0474; OR = 0.791). Additionally, in the recessive model (AA + AG versus GG), the genotype distribution of the rs10754557 SNP was significantly different between the cases and controls (*p* = 0.0344; *p*′ = 0.0352; OR = 1.277). We calculated the power of the genotype distribution using the PASS11 and found that the power of the genotype distribution of the rs3806265 SNP was 0.68420 and that of the rs10754557 SNP in the recessive model was 0.84836. However, there were no significant differences between the groups in either the genotype or allele frequencies for the SNPs rs8079034 (intron of NLRP1), rs11651270 (missense of NLRP1), rs11657747 (missense of NLRP1), rs878329 (31 kb 5′ of NLRP1), rs7512998 (intron of NLRP3), and rs10733113 (10 kb 3′ of NLRP3) (all *p* > 0.05).

### 3.3. Haplotype-Based Association Analysis

Haploview analysis revealed that there are 6 and 7 haplotype blocks existing on* NLRP1* and* NLRP3*, respectively, with the estimated frequency of more than 0.01. None of the 15 haplotypes were significantly associated with PsV risk (*p* > 0.05) (see Tables S3-S4 in the Supplementary Materials for the haplotypes information). As rs3806265 and rs10754557 SNPs in* NLRP3* are significantly associated with PsV, we also performed a haplotype-based association study specifically regarding the two SNPs. For the 4 blocks with the threshold of >0.01 (shown in Table 5), the most common was TA haplotype, and it was significantly associated with PsV (*χ*^2^ = 4.529; *p* = 0.033).

## 4. Discussion

Through this case-control study, we investigated the possible association between* NLRP1 *and* NLRP3* polymorphisms and PsV in Chinese Han population, by studying eight candidate SNPs. We observed that rs3806265 and rs10754557 in* NLRP3 *were associated with PsV.

Regarding PsV, a common and complex immune-mediated inflammatory disease, genetic factors have been shown to play a critical role in its pathogenesis [24], including numerous genetic variants related to innate immunity [25]. Given the key role of the NLRP family in innate immune regulation [18], we explored the association between SNPs in* NLRP1* and* NLRP3* and PsV.

In this study, rs3806265 and rs10754557 in* NLRP3* are associated with susceptibility to PsV. These results are partly consistent with those of the Swiss studies [9, 12]; however, there are some differences worth mentioning, namely, the lack of association of rs10733113 and the SNPs in* NLRP1* with PsV. However, an association study on Caucasians living in the UK showed that SNP rs3806265 in* NLRP3* is associated with psoriatic juvenile idiopathic arthritis [26], which is consistent with our finding. This discrepancy might be related to the heterogeneity of this complex polygenetic hereditary disease. The effect of susceptibility loci on the risk of psoriasis development may change under environment–gene interactions. Samples stability may also affect the results of the study.

In order to explore the function of rs3806265 and rs10754557, we used the HaploReg version 4.1 database (http://www.broadinstitute.org/mammals/haploreg) to identify the potential functional annotation of the risk alleles. We selected the position weight matrices from TRANSFAC and JASPAR databases to examine the effects of the risk allele on transcription factor binding using HaploReg. We examined transcriptional regulatory features such as DNase sensitivity, histone modifications, and transcription factor binding in the University of California Santa Cruz (UCSC) Encyclopedia of DNA Elements (ENCODE) database (https://www.encodeproject.org/). Available HaploReg data indicated that rs3806265 is located in intron region and histone marks (H3K4me1_Enh) are detected at the locus based on histone modification feature data in many immune-related cell lines (primary T helper memory cells from peripheral blood, primary T CD8+ naive cells from peripheral blood, and primary monocytes from peripheral blood), suggesting that this locus may function as an enhancer in the immune-related system. For rs10754557, we found that it is a trans-eQTL for* NLRP3* in whole blood according the HaploReg dataset, suggesting that the effects of rs10754557 and NLRP3 expression on disease developing risk may differ for individuals with different genotype.

It is worth mentioning that, in this study, most PsV patients are in relatively mild condition, which may be due to poor tolerance of patients with PsV, without waiting for the serious development of timely treatment. However, we did not find significant statistical differences between the* NLRP1* and* NLRP3* genotypes and the two groups of different severity of psoriasis, suggesting that both groups of different severity contribute to the overall significance. This is consistent with the findings of Europeans [9, 12].

This work was independently done by our team, including the sample collection, SNP genotyping, and statistical analysis. There are some limitations to our study. First, we analyze a single ethnic population in a single region, which inevitably leads to a certain degree of selection bias; the result may not be representative for other human races. Thus, to completely elucidate the roles of NLRP1 and NLRP3 in PsV, further studies in ethnically diverse populations are required to confirm or refute our findings. Second, with reference to previous literatures, in this study, we only included three clinical features to support the research samples. In future studies, we should consider more suspicious factors and make more detailed analysis so that our research can provide more value for the understanding of PsV. Additionally, subsequent functional evaluations are required to verify the functional prediction for the SNPs.

## 5. Conclusions

Taken together, this study investigated the association between* NLRP1* and* NLRP3 *polymorphisms and the risk of PsV in the Chinese Han population. Of the eight candidate SNPs investigated in* NLRP1* and* NLRP3*, two SNPs in* NLRP3 *(rs3806265 and rs10754557) were found to be significantly associated with PsV in the Chinese Han population. Overall, these findings supported the role of innate immunity in the pathogenesis of PsV and indicated that* NLRP3* polymorphisms may be a useful genetic marker or causative genetic factor in PsV development. Moreover, NLRP3 may be a potential therapeutic target for PsV.

## Figures and Tables

**Table 1:** Characteristics of the study participants.

Characteristic	Cases	Controls	*p*
Gender, *n* (%)			0.062
Male	332 (61.5%)	342 (55.9%)	
Female	208 (38.5%)	270 (44.1%)	
Age, mean ± SD	44.23 ± 12.44	45.47 ± 12.73	0.238
Age at onset, *n* (%)			
≤40 years	439 (81.3%)		
>40 years	101 (18.7%)		
PASI, *n* (%)			
≤10	446 (82.6%)		
>10	94 (17.4%)		
Family history, *n* (%)			
Yes	203 (37.6%)		
No	337 (62.4%)		

*n*, number; PASI, psoriasis area and severity index.

**Table 2:** Candidate SNPs location and frequency characteristics on NLRP1 and NLRP3 gene.

Gene	SNP	Chromosome position	Major/minor allele	Risk allele	Risk allele frequency	
					Case	Control
*NLRP1*	rs8079034	5509041	C/T	C	81.9%	80.9%
	rs11651270	5521757	T/C	C	24.3%	23.7%
	rs11657747	5541923	G/A	G/A	-	-
	rs878329	5649930	G/C	G	82.6%	81.5%
*NLRP3*	rs7512998	247419919	T/C	C	8.3%	7.4%
	rs3806265	247423034	T/C	T	56.5%	50.7%
	rs10754557	247435930	A/G	A	74.1%	69.1%
	rs10733113	247459055	G/A	A	6.1%	4.9%

**Table 3:** The single SNP association studies result of NLRP1 on the risk of PV.

Genotype	Cases (*n* = 540)	Controls (*n* = 612)	*p* value	Adjusted *p* value	Statistical model	*p*	*p*′	OR (95% CI)
rs8079034								
TT	16 (3.0%)	24 (3.9%)			Additive	0.8171	0.8374	
TC	164 (30.3%)	186 (30.4%)			Dominant	0.5288	0.6392	1.066
CC	360 (66.7%)	402 (65.7%)			Recessive	0.8040	0.8601	(0.792,1.435)
T/C	196/884	234/990	0.6732	0.7022	Heterozygous	0.9955	0.9998	
	18.1%/81.9%	19.1%/80.9%						
rs11651270								
TT	302 (55.9%)	360 (58.8%)			Additive	0.3893	0.3873	
TC	214 (39.6%)	214 (35.0%)			Dominant	0.4828	0.5006	1.032
CC	24 (4.4%)	38 (6.2%)			Recessive	0.3464	0.3666	(0.787,1.353)
T/C	818/262	934/290	0.8204	0.8320	Heterozygous	0.2480	0.2711	
	75.7%/24.3%	76.3%/23.7%						
rs11657747								
GG	526 (97.4%)	596 (97.4%)			Additive	-* *-	0.9999	
GA	14 (2.6%)	16 (2.6%)			Dominant	0.9869	0.9998	0.992
AA	0 (0)	0 (0)			Recessive	-* *-	0.9999	(0.357,2.753)
G/A	1066/14	1208/16	0.9869	0.9998	Heterozygous	0.9870	0.9999	
	98.7%/1.3%	98.7%/1.3%						
rs878329								
GG	364 (67.4%)	400 (65.3%)			Additive	0.8724	0.8946	
GC	164 (30.4%)	198(32.4%)			Dominant	0.60365	0.6660	0.931
CC	12 (2.2%)	14 (2.3%)			Recessive	0.9580	0.9999	(0.688,1.259)
G/C	892/188	998/226	0.62939	0.6359	Heterozygous	0.6089	0.6540	
	82.6%/17.4%	81.5%/18.5%						

*N*, number; *p*, model-based statistical *p* value; *p*′, *p* value adjusted by permutation; OR, odds ratio (we used the major allele as the reference allele to calculate the OR); 95% CI, 95% confidence interval.

**Table 4:** The single SNP association studies result of NLRP3 on the risk of PV.

Genotype	Cases (*n* = 540)	Controls (*n* = 612)	*p* value	Adjusted *p* value	Statistical model	*p*	*p*′	OR (95% CI)
rs7512998								
TT	450 (83.3%)	524 (85.6%)			Additive	0.3710	0.4540	
TC	90 (16.7%)	86 (14.1%)			Dominant	0.4488	0.4969	1.146
CC	0 (0.0%)	2 (0.3%)			Recessive	-* *-	0.9998	(0.745,1.762)
T/C	990/90	1134/90	0.5235	0.5760	Heterozygous	0.3846	0.4227	
	91.7%/8.3%	92.6%/7.4%						
rs3806265								
TT	166 (30.8%)	156 (25.5%)			Additive	0.1191	0.1157	
TC	278 (51.5%)	308 (50.3%)			Dominant	0.1614	0.1613	0.791
CC	96 (17.7%)	144 (24.2%)			Recessive	0.0595	0.0629	(0.627,0.998)
T/C	610/470	620/604	**0.0451** ^**∗**^	**0.0474** ^**∗**^	Heterozygous	0.7821	0.8072	
	56.5%/43.5%	50.7%/49.3%						
rs10754557								
GG	40 (7.4%)	52 (8.5%)			Additive	0.1054	0.1019	
GA	200 (37.0%)	274 (44.8%)			Dominant	0.6298	0.6516	1.277
AA	300 (55.6%)	286 (46.7%)			Recessive	**0.0344** ^**∗**^	**0.0352** ^**∗**^	(0.987,1.652)
G/A	360/800	378/846	0.0614	0.0655	Heterozygous	0.0595	0.0577	
	25.9%/74.1%	30.9%/69.1%						
rs10733113								
GG	474 (87.8%)	552 (90.2%)			Additive	-* *-	0.9999	
GA	66 (12.2%)	60 (9.8%)			Dominant	0.3540	0.4282	1.263
AA	0 (0.0%)	0 (0.0%)			Recessive	-* *-	0.9998	(0.760,2.100)
G/A	1014/66	1164/60	0.3540	0.4282	Heterozygous	0.3540	0.4282	
	93.9%/6.1%	95.1%/4.9%						

*N*, number; *p*, model-based statistical *p* value; *p*′, *p* value adjusted by permutation; OR, odds ratio (we used the major allele as the reference allele to calculate the OR); 95% CI, 95% confidence interval; *∗* indicates the significant association.

**Table 5:** The haplotype-based association study of rs3806265 and rs10754557 SNPs in NLRP3 gene.

Haplotype	Freq.	Case, control ratio counts	Case, control freqs.	*χ* ^2^	*p*
TA	50.7%	291.9 : 248.1, 292.4 : 319.6	54.1%, 47.8%	4.529	0.0333
CG	25.9%	126.9 : 413.1, 171.4 : 440.6	23.5%, 28.0%	3.031	0.0817
CA	20.7%	108.1 : 431.9, 130.6 : 481.4	20.0%, 21.3%	0.307	0.5797
TG	2.7%	13.1 : 526.9, 17.6 : 594.4	2.4%, 2.9%	0.227	0.6335

Freq., frequency; *χ*^2^, chi-square;  *p*, *p* value.

## Data Availability

According to the informed consent signed by the volunteers, the aggregated SNP genotype frequency and allele frequency data in the manuscript can be available freely to the academic community. The SNP genotype and the clinical information can be obtained by e-mail after security audit.

## Supplementary Materials

Supplementary Materials Tables S1-S2: primers and probes displays for PCR and imLDR. Tables S3-S4: haplotype analysis. Table S1: primers used for the genotyping of NLRP1 and NLRP3. Table S2: iMLDR probe sequences. Table S3: the haplotype-based association study of NLRP1 gene polymorphisms. Table S4: the haplotype-based association study of NLRP3 gene polymorphisms.
