# Supplementary material for: Association of NLRP1 and NLRP3 Polymorphisms with Psoriasis Vulgaris Risk in the Chinese Han Population
Source: Biomed Res Int. 2018 Apr 3;2018:4714836. doi: 10.1155/2018/4714836 (PMC5903344; doi:10.1155/2018/4714836)
Supplement: Supplementary Materials — Tables S1-S2: primers and probes displays for PCR and imLDR. Tables S3-S4: haplotype analysis. Table S1: primers used for the genotyping of NLRP1 and NLRP3. Table S2: iMLDR probe sequences. Table S3: the haplotype-based association study of NLRP1 gene polymorphisms. Table S4: the haplotype-based association study of NLRP3 gene polymorphisms. [file 4714836.f1.docx]

**Supplementary material**

Table S1 Primers used for the genotyping of NLRP1 and NLRP3.

| Gene | SNP | PCR primer (5’-3’)  Forward | PCR primer (3’-5’)  Reverse | Product  length, bp |
| --- | --- | --- | --- | --- |
| NLRP1 | rs8079034 | ATTCATGCCCACGCAGACAAA | ACAGGTTAGCCCCAGGCAGGTA | 250 |
|  | rs11651270 | CGCAGGGCATTATGGATCATTT | TTACAGGGGGCCATGTGGA | 166 |
|  | rs11657747 | TAGGCTCCTCCCACTCCCACAA | TGAGAGCCAACCAGACCCTGAC | 234 |
|  | rs878329 | TTTACTTTCCCGGGCTGCATCA | CCAACCACCAACATGAGACCAG | 224 |
| NLRP3 | rs7512998 | CGGAATTCCCTTCCTTTAAAAAAGACT | TTGGTGGGAAATGGTGCAGA | 282 |
|  | rs3806265 | AACACATGCTTGGCAGGTGGA | TGGGATTCGAAACACGTGCATTA | 256 |
|  | rs10754557 | TCCACCAAATGGAAAAGGAAATCAG | CACCCATGAAGACTTACCCCAGTT | 350 |
|  | rs10733113 | GCATGAGACCTGAACTGGCTTCA | GCACTACTTCTTGCGGCCTGTC | 321 |

Table S2 iMLDR probe sequences.

| Gene | Snp Allele | Primer (5’-3’) | LDR product |
| --- | --- | --- | --- |
| NLRP1 | rs8079034_modify: | GTTAGGTAAGGACCTTTGTCTGCGT TTTTTTTTTTT | 63.04 |
|  | rs8079034_C: | TCTCTCGGGTCAATTCGTCCTTAATGCGGGGCCATCTAGTCG | 70.42 |
|  | rs8079034_T: | TGTTCGTGGGCCGGATTAGT AATGCGGGGCCATCTAGTCA | 69.02 |
| NLRP1 | rs11651270_modify: | YGGCCCACTTTAAAGAGGAGGGTTTTTTTTTTTTTT | 65.64 |
|  | rs11651270_C: | TTCCGCGTTCGGACTGATATTGTGGACACATCCCTGTTCCTAG | 66.52 |
|  | rs11651270_T: | TACGGTTATTCGGGCTCCTGTTGTGGACACATCCCTGTTCCCAA | 66.83 |
| NLRP1 | rs11657747_modify: | TGAGCACATTGAAGCTCAGGTCCTTTTTTTTT | 66.05 |
|  | rs11657747_A: | TGTTCGTGGGCCGGATTAGTGGTGTTTGGCTCCAGCATGCA | 67.74 |
|  | rs11657747_G: | TCTCTCGGGTCAATTCGTCCTTGGTGTTTGGCTCCAGCATACG | 69.26 |
| NLRP1 | rs878329_modify: | TCATCTCCAACSCAGTCATGAGGTTTTTTTTTTTTTTT | 65.70 |
|  | rs878329_C: | TGTTCGTGGGCCGGATTAGTCAACCCCCAATTCAACTTTTGAGC | 67.44 |
|  | rs878329_G: | TCTCTCGGGTCAATTCGTCCTTCAACCCCCAATTCAACTTTTGAGG | 67.32 |
| NLRP3 | rs7512998_modify: | TGGGGTATATACTCAGAAGTGAAATTTCTGTTTTTTTTTTTTTTTTTTTTTT | 63.70 |
|  | rs7512998_C: | TCTCTCGGGTCAATTCGTCCTTTCTCTTCAAGACCCTGCTTTGAATTGTC | 66.97 |
|  | rs7512998_T: | TGTTCGTGGGCCGGATTAGTTCTCTTCAAGACCCTGCTTTGAATTGTT | 66.46 |
| NLRP3 | rs3806265_modify: | TGTTTTGAAACTAGGAGTGCAGAAATGTTTTTTTTTTTTTTTTTTT | 63.80 |
|  | rs3806265_C: | TCTCTCGGGTCAATTCGTCCTTTTGGCAGGTGGACAGCAGATGC | 69.14 |
|  | rs3806265_T: | TGTTCGTGGGCCGGATTAGTTTGGCAGGTGGACAGCAGACGT | 66.54 |
| NLRP3 | rs10754557_modify: | GTGAGAGACATGACTGACATTCTGCCTTTTTTTTTTTTT | 65.61 |
|  | rs10754557_A: | TGTTCGTGGGCCGGATTAGTTTGTCCATGGTGGAGCGAGA | 66.07 |
|  | rs10754557_G: | TCTCTCGGGTCAATTCGTCCTTTTGTCCATGGTGGAGCGAGG | 67.67 |
| NLRP3 | rs10733113_modify 1: | ATCAACAACAACAACAACAAAAAGCCTTTTTTTTTTTTTTTTTTTT | 64.31 |
|  | rs10733113_modify 2: | ATCAACAACAACAACAACAAACCCTCTTTTTTTTTTTTTTTTTTTT | 64.33 |
|  | rs10733113_G: | TTCCGCGTTCGGACTGATATCCATGTGAAAAATATAATAGGGTGCTGAGACAAGAC | 70.17 |
|  | rs10733113_A: | TACGGTTATTCGGGCTCCTGTCCATGTGAAAAATATAATAGGGTGCTGAGACAACAT | 70.29 |

Table S3 The haplotype-based association study of NLRP1 gene polymorphisms.

| Haplotype | Frequency | Case, Control Ratio Counts | Case,Control Frequencies | Chi Square | P Value |
| --- | --- | --- | --- | --- | --- |
| CTGG | 56.9% | 622.0 : 458.0, 689.8 : 534.2 | 57.6%, 56.4% | 0.177 | 0.6740 |
| TTGG | 14.9% | 160.2 : 919.8, 183.0 : 1041.0 | 14.8%, 15.0% | 0.004 | 0.9515 |
| CCGC | 12.9% | 144.8 : 935.2, 153.0 : 1071.0 | 13.4%, 12.5% | 0.205 | 0.6508 |
| CCGG | 7.3% | 81.4 : 998.6, 86.0 : 1138.0 | 7.5%, 7.0% | 0.111 | 0.7386 |
| CTGC | 4.2% | 35.8 : 1044.2, 61.0 : 1163.0 | 3.3%, 5.0% | 1.984 | 0.1590 |
| TCGG | 2.1% | 19.8 : 1060.2, 29.4 : 1194.6 | 1.8%, 2.4% | 0.438 | 0.5079 |

Table S4 The haplotype-based association study of NLRP3 gene polymorphisms.

| Haplotype | Frequency | Case, Control Ratio Counts | Case,Control Frequencies | Chi Square | P Value |
| --- | --- | --- | --- | --- | --- |
| TTAG | 42.3% | 481.4 : 598.6, 492.4 : 731.6 | 44.6%, 40.2% | 2.222 | 0.1361 |
| TCGG | 23.2% | 223.6 : 856.4, 310.4 : 913.6 | 20.7%, 25.4% | 3.491 | 0.0617 |
| TCAG | 19.9% | 208.0 : 872.0, 250.0 : 974.0 | 19.3%, 20.4% | 0.242 | 0.623 |
| CTAG | 6.2% | 73.6 : 1006.4, 70.2 : 1153.8 | 6.8%, 5.7% | 0.576 | 0.448 |
| TCGA | 2.7% | 30.4 : 1049.6, 31.8 : 1192.2 | 2.8%, 2.6% | 0.046 | 0.8308 |
| TTGG | 2.0% | 18.2 : 1061.8, 29.0 : 1195.0 | 1.7%, 2.4% | 0.686 | 0.4076 |
| TTAA | 1.5% | 10.8 : 529.2, 6.9 : 605.1 | 2.0%, 1.1% | 1.441 | 0.2300 |
